# A peritoneal effluent sequencing assay that removes environmental DNA contamination in peritoneal dialysis patients

**DOI:** 10.1093/ckj/sfaf358

**Published:** 2025-12-26

**Authors:** Liz-Audrey Kounatse Djomnang, Vesh Srivatana, Omary Mzava, Emma Belcher, Lars F Westblade, Darshana M Dadhania, Carol Li, Iwijn De Vlaminck, John R Lee

**Affiliations:** Meinig School of Biomedical Engineering, Cornell University, Ithaca, NY, USA; The Rogosin Institute, New York, NY, USA; Division of Nephrology and Hypertension, Department of Medicine, Weill Cornell Medicine, New York, NY, USA; Meinig School of Biomedical Engineering, Cornell University, Ithaca, NY, USA; Meinig School of Biomedical Engineering, Cornell University, Ithaca, NY, USA; Department of Pathology and Laboratory Medicine, Weill Cornell Medicine, New York, NY, USA; Division of Nephrology and Hypertension, Department of Medicine, Weill Cornell Medicine, New York, NY, USA; Department of Transplantation Medicine, New York Presbyterian Hospital–Weill Cornell Medical Center, New York, NY, USA; Division of Nephrology and Hypertension, Department of Medicine, Weill Cornell Medicine, New York, NY, USA; Meinig School of Biomedical Engineering, Cornell University, Ithaca, NY, USA; Division of Nephrology and Hypertension, Department of Medicine, Weill Cornell Medicine, New York, NY, USA; Division of Renal-Electrolyte and Hypertension, Department of Medicine, Perelman School of Medicine, University of Pennsylvania, Philadelphia, PA, USA

To the Editor,

Newer DNA sequencing techniques have the potential to identify microbial DNA in peritoneal dialysis (PD) patients with peritonitis and without peritonitis. However, given the potential low biomass of microbial DNA in peritoneal effluent, introduction of contaminant DNA during the sequencing process can lead to errors in microbial identification. We have developed Sample-Intrinsic microbial DNA Found by Tagging and Sequencing (SIFT-seq), a metagenomic sequencing assay that is robust to contamination [1]. The key concept for SIFT-seq is the chemical labeling of DNA by bisulfite salt treatment prior to sequencing, and any DNA without the chemical signature can be identified and removed [1]. SIFT-seq was shown to identify microbes in blood and urine specimens while removing environmental DNA contamination [1]. In this study, we assess its utility in PD patients with culture-confirmed peritonitis and culture-negative peritonitis.

We recruited 31 PD patients who provided a total of 35 peritoneal effluent specimens. The Weill Cornell IRB approved this protocol (1 604 017 181) and all patients provided written informed consent. Full details of SIFT-seq can be found in Mzava *et al*. [1], the Supplementary data and Fig. 1A. Briefly, we collected peritoneal effluent specimens from subjects in the PD clinic or the hospital. Bisulfite treatment was performed on the peritoneal effluent supernatant using either Lightning-conversion [1] or methylation-direct kit (Zymo Research, Irvine, CA, USA). Cell-free DNA (cfDNA) libraries were sequenced on an Illumina Nextseq 2000 (Illumina, San Diego, CA, USA). cfDNA sequences without bisulfite conversion (contaminant cfDNA) were removed bioinformatically and cfDNA tissue-of-origin analysis were performed as described in the Supplementary data.

**Figure 1:**
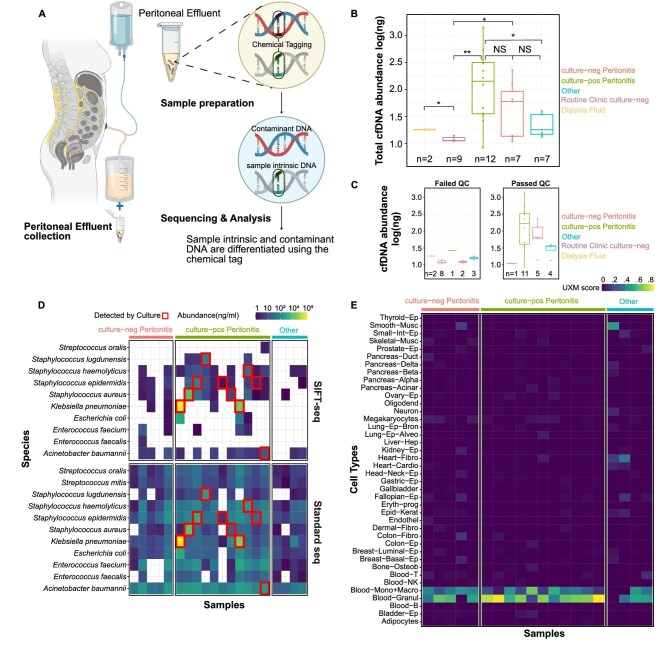
SIFT-seq experimental design and results in peritoneal dialysis patients. (**A**) Overview of SIFT-seq assay. Peritoneal effluent is collected from a PD patient. The peritoneal effluent supernatant is collected, and the cfDNA is subjected to bisulfite treatment which converts cytosine to uracil (i.e. chemical tagging). After sequencing, sample intrinsic DNA and contaminant DNA (introduced after chemical tagging) can be differentiated bioinformatically. (**B**) Peritoneal effluent specimens by total cfDNA abundance. Peritoneal effluent specimens by groups are represented on the x-axis and cfDNA abundance by log scale is on the y-axis. (**C**) Peritoneal effluent specimens by QC status. Peritoneal effluent specimens by group are represented on the x-axis and cfDNA abundance (ng/mL) by log scale is on the y-axis. Passing QC was defined as at least 10 million reads post trimming, 10% mapping efficiency and 80% conversion efficiency. The left graph shows the peritoneal effluent specimens that failed QC and the right graph shows the peritoneal effluent specimens that passed QC. The cfDNA abundance was overall higher in the peritoneal effluent specimens that passed QC than those that failed QC. (**D**) Detection of specific pathogenic bacteria by SIFT-seq and standard sequencing without bisulfite treatment. Individual samples are represented on the x-axis by group and specific bacterial species are on the y-axis. The abundances detected by the sequencing technology are represented by color in the individual boxes with the scale in the legend. Standard sequencing leads to detection of many bacteria at low levels (bottom graph) but SIFT-seq leads to more specific detection of bacteria (top graph). Importantly, SIFT-seq detects the bacteria identified in routine clinical culture in most cases of culture-positive bacterial peritonitis. In one peritonitis case of *Acinetobacter junii*, *Acinetobacter baumannii* cfDNA was detected. (**E**) Cell and tissue of origin analysis for peritoneal effluent specimens by group. Individual samples are represented on the x-axis by group and cell/tissue categories are on the y-axis. The cell/tissue abundances are represented by color in the individual boxes with the scale in the legend. Methylated DNA from monocytes, macrophages and granulocytes were detected in most of the peritoneal effluent specimens in the culture-neg peritonitis and culture-pos peritonitis groups. UXM score: U, mostly unmethylated; M, mostly methylated; or X, mixed.

Among the 35 peritoneal fluid specimens, 12 were from 12 PD patients with culture-confirmed bacterial peritonitis (culture-pos peritonitis); 7 were obtained from 7 PD patients with culture-negative peritonitis (culture-neg peritonitis); 9 were obtained from 8 PD patients without symptoms in the PD clinic and had negative peritoneal effluent culture (routine clinic culture-neg); and 7 were obtained from PD patients with a range of pathologies (2 with fungal peritonitis, 2 with abdominal pain without evidence of peritonitis, 2 with hypertensive crisis and 1 with prior peritonitis) (other). PD patients may have contributed specimens across groups. Two specimens were taken directly from Baxter-manufactured solutions (Baxter International, Deerfield, IL, USA) as controls (neg control). Demographic/clinical data are presented in Supplementary data, Table S1.

Twenty-one of the 35 peritoneal effluent specimens passed quality control (QC) with at least 10 million reads post-trimming, 10% mapping efficiency and 80% conversion efficiency. Importantly, eight of the nine peritoneal effluent specimens from the routine clinic culture-neg group and both specimens from the neg control group did not pass QC, suggesting a lower limit to the utility of SIFT-seq on low biomass specimens. In contrast, 11 of 12 specimens from the culture-pos peritonitis group passed QC and 5 of 7 specimens from the culture-neg peritonitis passed QC (Fig. 1B and C).

Among the 21 specimens that passed QC, common microbes causing peritonitis were abundantly detected in all specimens prior to computational removal of contaminant cfDNA (Fig. 1D). However, after filtering out contaminant DNA, there was significant reduction in microbial noise and notably the presumed causative agent of culture-pos peritonitis cases was detected in all cases except for one peritonitis case of *Acinetobacter junii*, but *Acinetobacter baumannii* cfDNA was detected (Fig. 1D). Importantly, in two of the culture-pos peritonitis cases, specimens were taken after antibiotic administration, suggesting the use of SIFT-seq in PD patients who may have already been treated for peritonitis. Tissue-of-origin analysis revealed that the majority of cfDNA originates from monocytes, macrophages and granulocytes in culture-pos and culture-neg peritonitis (Fig. 1E). Limitations of the study include the small sample size, the lack of comparison with 16S rRNA gene sequencing and the exploratory nature of SIFT-seq on peritoneal effluent, restricting generalizability of the conclusions. Despite these limitations, SIFT-seq may be a novel technique for detecting microbes in PD patients with and without peritonitis.

## Supplementary Material

sfaf358_Supplemental_File

## Data Availability

Sequencing data including raw fastq files, demographic and clinical data will be made available in the database of Genotypes and Phenotypes (dbGaP) phs002251.v1.p1. Local institutional review board approval will be needed to access the data.
