# Supplementary material for: A peritoneal effluent sequencing assay that removes environmental DNA contamination in peritoneal dialysis patients
Source: Clin Kidney J. 2025 Dec 26;19(1):sfaf358. doi: 10.1093/ckj/sfaf358 (PMC12780757; doi:10.1093/ckj/sfaf358)
Supplement: sfaf358_Supplemental_File [file sfaf358_supplemental_file.docx]

**Supplementary Information**

**A Peritoneal Effluent Sequencing Assay that Removes Environmental DNA Contamination in Peritoneal Dialysis Patients**

Liz-Audrey Kounatse Djomnang^1^, Vesh Srivatana^2,3#^, Omary Mzava^1^, Emma Belcher^1^, Lars F. Westblade^4^, Darshana M. Dadhania^3,5^, Carol Li^3^, Iwijn De Vlaminck* ^1^, John R. Lee* ^3,6^

1. Meinig School of Biomedical Engineering, Cornell University, Ithaca, NY, USA
2. The Rogosin Institute, New York, NY, USA
3. Division of Nephrology and Hypertension, Department of Medicine, Weill Cornell Medicine, New York, NY, USA
4. Department of Pathology and Laboratory Medicine, Weill Cornell Medicine, New York, NY, USA
5. Department of Transplantation Medicine, New York Presbyterian Hospital–Weill Cornell Medical Center, New York, NY, USA
6. Division of Renal-Electrolyte and Hypertension, Department of Medicine, Perelman School of Medicine, University of Pennsylvania, Philadelphia, PA, USA

* co-senior and corresponding authors

Correspondence to: Dr. Iwijn de Vlaminck, Meinig School of Biomedical Engineering, Cornell University, Weill Hall Room 301, Ithaca, NY, 14853, email address: vlaminck@cornell.edu, or Dr. John R. Lee, Division of Nephrology and Hypertension, Department of Medicine, Weill Cornell Medicine, 525 E. 68^th^ Street Box 3, New York, NY 10065, email address: jrl2002@med.cornell.edu or Division of Renal, Electrolyte, and Hypertension, Department of Medicine, Perelman School of Medicine, University of Pennsylvania, 3400 Spruce Street Floor 1, Philadelphia, PA, USA, email address: john.lee4@pennmedicine.upenn.edu

# Current Affiliation, Division of Nephrology, Department of Medicine, Columbia University Vagelos College of Physicians & Surgeons, New York, NY, USA

**Supplementary Table 1**

Characteristics of the Peritoneal Dialysis Cohort

|  |  |  |  |
| --- | --- | --- | --- |
|  |  | PD Cohort (n = 31) |  |
|  | Characteristics | n, % or mean±SD |  |
|  | Age in years (mean±SD) | 55±14 |  |
|  | Female Sex | 21 (68%) | ± |
|  | Race |  |  |
|  | White | 9 (29%) |  |
|  | Black or African American | 14 (45%) |  |
|  | Asian | 2 (6%) |  |
|  | American Indian or Alaska Nation | 1 (3%) |  |
|  | Other/Declined | 5 (16%) |  |
|  | Ethnicity |  |  |
|  | Hispanic or Latino | 6 (19%) |  |
|  | Not Hispanic or Latino | 22 (71%) |  |
|  | Declined | 3 (10%) |  |
|  |  |  |  |
|  | History of Hypertension | 29 (94%) |  |
|  | History of Diabetes Mellitus | 13 (42%) |  |
|  | Cause of End Stage Kidney Disease |  |  |
|  | Hypertension | 6 (19%) |  |
|  | Diabetes Mellitus | 4 (13%) |  |
|  | Hypertension/Diabetes Mellitus | 5 (16%) |  |
|  | Polycystic Kidney Disease | 3 (10%) |  |
|  | Systemic Lupus Erythematosus | 2 (6%) |  |
|  | Other/Unknown | 11 (35%) |  |
|  |  |  |  |
|  | Type of Peritoneal Dialysis |  |  |
|  | CAPD | 6 (19%) |  |
|  | CCPD | 25 (81%) |  |
|  | Time on Peritoneal Dialysis |  |  |
|  | in years (mean±SD) | 2.5±1.8 |  |
|  |  |  |  |

CAPD, continuous ambulatory peritoneal dialysis; CCPD, continuous cycling peritoneal dialysis; SD, standard deviation; PD, peritoneal dialysis

**Supplementary Table 2**

Distribution of samples across bisulfite conversion techniques

| Bisulfite conversion method | Number of sequenced samples |
| --- | --- |
| Methylation direct (Passed QC) | 5 |
| Lightning conversion (Passed QC) | 16 |
| Both techniques (Failed QC) | 14 |

**Supplementary Table 3**

Distribution of sequencing parameters across samples

| Parameter | Median (Q1, Q3) | Mean ± SD |
| --- | --- | --- |
| Total number of reads | 48973612 (39937108, 67480779) | 61603863 ± 59098410 |
|  |  |  |
| cfDNA yield |  |  |
| Total cfDNA yield (ng/ml) | 14.27 (11.6, 38.2) | 81.8 ± 208.5 |
| Peritonitis (ng/ml) | 36.5 (15.9, 195) | 180 ± 333 |
| Culture neg (ng/ml) | 37.1 (13.5, 115) | 68.2 ± 74.3 |
| Other (ng/ml) | 18.2 (11.4, 14.1) | 18.2 ± 0.831 |
|  |  |  |
| Bisulfite conversion efficiency |  |  |
| Total (%) | 85.77 (41.7, 97.8) | 70.6 ± 32.5 % |
| Passed QC(%) | 98.2 % (96.7, 99.2) | 96.7 ± 4.8 % |
| Failed QC (%) | 46.1 % (23.2, 78.9) | 51.5 ± 30.9 % |

**Supplementary Methods**

**Description of the study cohort and sample collection**

Under Weill Cornell IRB-approved protocol 1604017181, we recruited 31 PD patients who provided a total of 35 peritoneal effluent specimens. Peritoneal effluent specimens were collected by drainage of fluid from the peritoneal catheter in PD patients. Peritoneal effluent specimens were almost exclusively from Baxter manufactured solutions (Baxter, Deerfield, Illinois, USA). The specimen was subsequently centrifuged at 2,000 × g for 30 minutes, and 1-mL aliquots of peritoneal effluent supernatants were collected and stored at -80 °C.

Among the 35 peritoneal effluent specimens, 12 were from PD patients with culture-confirmed bacterial peritonitis (culture-pos peritonitis group); 7 from PD patients with culture-negative peritonitis (culture-neg peritonitis group); 9 from PD patients without symptoms and had negative peritoneal fluid culture (routine clinic group); and 7 from PD patients with a range of pathologies (2 with fungal peritonitis, 2 with abdominal pain without evidence of peritonitis, 2 with HTN crisis, and 1 with prior peritonitis) (Other group). Two specimens were taken directly from Baxter manufactured solutions, never instilled into a patient as controls (neg control).

**Rationale and Overview of SIFT-seq**

Environmental DNA contamination has been increasingly recognized as a significant challenge in low-biomass sequencing studies. Several reports in recent years have described microbial signatures in samples traditionally considered sterile, such as amniotic fluid[1,2], and plasma[3]. Many of these findings remain disputed, as the identified taxa were likely introduced through contamination rather than representing true biological signals[4–6].

The primary sources of such contamination are well characterized and include laboratory reagents, consumables, equipment, personnel, and cross-sample contamination [7–10]. While the magnitude of the problem varies by sample type and biomass, the consequences can be substantial: misinterpretation of microbiome data, spurious associations in clinical studies, and even compromised integrity of forensic DNA evidence[11].

Previous studies have shown that most sequencing contaminants originate from reagents, tools, and laboratory environments rather than the biological sample itself [7–10]. In our earlier work on SIFT-seq [12], we specifically used positive controls (ΦX174 DNA and mock microbial communities) to demonstrate the impact of contamination across biomass levels, and how well SIFT-seq performed in reducing the impact of environmental contamination. These experiments showed that lower-biomass samples are more prone to contamination and that SIFT-seq reliably recovers original sequences while minimizing contaminant contributions.

Furthermore, during SIFT-seq, DNA is subjected to the spontaneous deamination of cytosines to uracil (C→U) directly in the biofluid before DNA isolation. Spontaneous hydrolytic deamination of cytosine to uracil in double-stranded DNA is rare (k ≈ 7 × 10⁻¹³ s⁻¹ at 37 °C), corresponding to only ~70–500 C→U events per human genome per day; over the ≤24 h between collection and bisulfite tagging, a 150-bp cfDNA fragment (with ~20–30 cytosines) therefore has an expected probability ≪1 (≈10⁻⁶) of acquiring even a single uracil via spontaneous deamination [13–15]; multi-site conversion across a molecule is vanishingly unlikely.

In SIFT-seq, intrinsic molecules are intentionally and pervasively converted by bisulfite, whereas contaminants introduced after tagging retain many cytosines and are removed by our read-level filter, which excludes any read with >3 cytosines or any CpG (as described in our prior SIFT-seq paper [12]. Consequently, rare spontaneous C→U events cannot make an untagged contaminant appear tagged, and they do not meaningfully affect tissue-of-origin or microbiome analyses. Libraries with poor conversion or mapping show a characteristic shift in cytosine-fraction distributions and are excluded.

**Cell-free DNA processing**

Bisulfite treatment was performed on peritoneal supernatant using either the Lightning-conversion as demonstrated in [12] or the methylation-direct kit (Zymo Research, products #5030 and #5020, Irvine, California, USA), which utilizes a chemistry optimized for shorter DNA molecules such as cfDNA. Cell-free DNA (cfDNA) was isolated using QIAamp Circulating Nucleic Acid Kit (QIAGEN, Cat# 55114, Germantown, Maryland, USA), and library preparation was conducted with SRSLY Pico-Plus DNA NGS Library Preparation Base Kit (Claret Biosciences, product #CBS-K150B, Santa Cruz, California, USA).

**SIFT-seq in peritoneal effluent**

SIFT seq was performed on 35 specimens using methods described in [12], and a modified version of the same protocol. All samples (n = 19) with low conversion rates after processing via the original SIFT-seq protocol [12] were processed again using a modified version of the same protocol (see Supplemental Table 2 for the distribution of samples across each method). All samples were processed alongside internal controls to ensure assay performance. Naked DNA controls were used to assess extraction efficiency, resulting in ~80% DNA recovery. As a practical negative control, we sequenced two Baxter manufactured dialysis fluids, which yielded very low DNA and insufficient reads for downstream analysis. For downstream tissue-of-origin and microbiome analyses, all included samples achieved ≥80% bisulfite conversion efficiency. Supplemental Table 3 has further details of sequencing quality metrics: total numbers of reads, cfDNA yield per group, and bisulfite conversion efficiency.

The modified SIFT-seq protocol is as described below.

An aliquot of 200 µL of peritoneal fluid was centrifuged at 20,000 × g for 10 min at 10 °C to pellet cellular debris. The supernatant was transferred to a new microplate, each well containing 20 μL of supernatant. 130 μL of sodium metabisulfite solution (Zymo Research, product #5003-1, Irvine, California, USA). The sodium metabisulfite solution was prepared according to the manufacturer’s recommendation using the CT conversion reagent (Zymo Cat# D5003-1, Irvine, California, USA), M-Solubilization Buffer (Zymo Cat# D5021-7, Irvine, California, USA), M-Dilution Buffer (Zymo Cat# D5002-2, Irvine, California, USA), and M-Reaction Buffer (Zymo Cat# D5021-8, Irvine, California, USA). After a quick vortex and spin, the resulting mixture was incubated in a thermocycler at 98 °C for 10 min, and 64 °C for 3.5 H. Following the incubation, fluid from the wells was transferred to a 50 ml conical tube, and the volume was completed to 1 mL by adding 1x PBS. cfDNA extraction was performed using the QIAamp Circulating Nucleic Acid Kit using the 1 mL plasma protocol (Qiagen, product #55114. Germantown, Maryland, USA). DNA was eluted to 150µL and transferred to a Zymo spin IC column (Zymo Cat# C1004-50, Irvine, California, USA) containing 600 µL of M-binding buffer (Zymo Cat# D5001-3, Irvine, California, USA). After mixing both DNA and binding buffer, the column was spun at full speed (14,000 x g) for 30 seconds and washed with 100 μL of M-Wash buffer (Zymo Research, product #5001-6, Irvine, California, USA). 200 µL M-Desulphonation buffer (Zymo Research, product #5001-5, Irvine, California, USA) was added to the columns for 20 min, followed by two washes with 200 µL M-Wash buffer. DNA was then eluted with the M-Elution Buffer (Zymo Cat# D5001-6, Irvine, California, USA) according to the manufacturer's recommendations, and single-stranded library preparation was performed using the SRSLY Pico-Plus DNA NGS Library Preparation Base Kit (Claret Biosciences, product #CBS-K150B, Santa Cruz, California, USA), and KAPA HiFi HotStart Uracil+ ReadyMix (2X) (Roche Cat# 7959052001, Basel, Switzerland) according to methods described in [12]. Libraries were then sequenced on an Illumina sequencer.

A step-by-step modified SIFT-seq protocol is provided below.

**Modified SIFT-seq in Peritoneal fluid; Step-by-Step protocol.**

**Step 1: Bisulfite Conversion**

1. Preheat a thermal cycler. Program 98°C for 10 min and 64°C for 3.5 hours.
2. Set the water bath to 60°C.
3. Thaw biofluid aliquots on ice and spin down for 10 min at 14,000 RPM and 10°C.
4. Transfer 20 µL of the supernatant to different wells on a microplate.
5. Add 130ul CT Conversion reagent to each well.
6. Incubate in the 98°C thermocycler for 10 minutes.
7. Incubate in the 64°C thermocycler for 3.5 hours
8. Immediately proceed to the next step (or store in 4°C for up to 20 hours)

**Step 2: cfDNA Extraction and Desulphonation**

1. Collect the biofluid-CT reagent mixture from the microplate and complete it to 1ml with 1x PBS.
2. Add 100 µL of Proteinase K into a 50 mL centrifuge tube.
3. Add the mixture from 1 to the 50 mL tube containing Proteinase K.
4. Add 0.8 mL Buffer ACL (containing 1ug of carrier RNA). Pulse vortex 30 seconds.
5. Incubate at 60°C for 30 minutes.
6. Place the tube back on the lab bench and unscrew the cap.
7. Add 1.8 mL Buffer ACB to the lysate. Pulse vortex 15-30 seconds.
8. Incubate on ice for 5 minutes.
9. Prepare the manifold.
10. Add the lysate-buffer ACB mixture to the tube extender and turn on the vacuum. Drain the column.
11. Apply 600 µL ACW1. Drain the column.
12. Apply 750 µL ACW2. Drain the column.
13. Apply 750 µL 96-100% ethanol. Drain the column.
14. Transfer the column to a collection tube and dry at 56°C for 10 min.
15. Transfer to an elution tube.
16. Add 155 µL of Buffer AVE to the center of the column. Close the lid and incubate for 3 minutes at room temperature.
17. Centrifuge at 14,000 RPM for 1 minute.
18. Add 600 µL of M-Binding Buffer to a Zymo-Spin IC Column assembled in a collection tube.
19. Load the sample (150 µL from step 16) into the Zymo-Spin IC Column containing the M-Binding Buffer. Close the cap and mix by inverting the column several times.
20. Centrifuge at full speed (14,000 x g) for 30 seconds. Discard the flow-through.
21. Add 100 µL of M-Wash Buffer to the column. Centrifuge at full speed for 30 seconds.
22. Add 200 µL of M-Desulphonation Buffer to the column and let it stand at room temperature (20-30°C) for 20 minutes.
23. After the incubation, centrifuge at full speed for 30 seconds.
24. Add 200 µL of M-Wash Buffer to the column. Centrifuge at full speed for 30 seconds.
25. Repeat this wash step.
26. Place the column into a 1.5 mL microcentrifuge tube and add 32 µL of M-Elution Buffer directly to the column matrix. Centrifuge at 30 seconds at full speed to elute the DNA.
27. Measure ssDNA abundance using Qubit ssDNA Assay Kit (Invitrogen Cat# Q10212, Waltham, MA) according to the manufacturer’s recommendations.

Samples can be stored at -20°C.

**Step 3: Library preparation and sequencing**

Libraries are prepared with SRSLY Pico-Plus DNA NGS Library Preparation Base Kit (Claret Biosciences, Cat# CBS-K250B-24, Santa Cruz, California, USA), and KAPA HiFi HotStart Uracil+ ReadyMix (2X) (Roche Cat# 7959052001, Basel, Switzerland) as described in [12].

cfDNA libraries can be sequenced on an Illumina NextSeq 2000 sequencer (Illumina, San Diego, CA) with a 2 × 50 bp read length.

**Alignment to the human genome**

Adapter and low-quality bases from the reads were trimmed using BBDuk [16] (BBDuk V38.4634,–entropy = ‘0.25’–maq = ‘10’ -Xmx1g tbo tpe) and aligned to the C-to-T and G-to-A converted human genome using Bismark (Bismark-0.22.1 [17] ,–unmapped,–quiet). PCR duplicates were removed using Bismark.

**Depth of coverage**

The depth of sequencing was measured by summing the depth of coverage for each mapped base pair on the human genome after duplicate removal, and dividing by the total length of the human genome (hg19, without unknown bases).

**Removing unconverted molecules**

Aligned BAM files are filtered to remove unconverted molecules using the Bismark [17] (Bismark-0.22.1) alignment package with default parameters.

**Bisulfite conversion efficiency**

We estimated bisulfite conversion efficiency by quantifying the rate of C[A/T/C] methylation in human-aligned reads (using MethPipe [18] V3.4.3), which are rarely methylated in mammalian genomes.

**Pre-processing of the unmapped reads**

Reads originating from the Phix genome were removed from the host unmapped reads using Bowtie 2 [19] (Bowtie 2.4.3,–local,–very-sensitive-local,–un-conc). Read IDs from the remaining reads were used to subset paired-end reads from the original FASTQ files. Adapter trimming and read quality filtering were performed using BBDuk [16] (BBDuk V38.46, maq = 32). Remaining reads were deduplicated using samtools (samtools V1.14 [20]) and merged using FLASH2 [21] (-q -M75 -O). K-mer decontamination to remove human reads was then performed using BBDuk (BBDuk V38.46, k = 50, prealloc = t), and the obtained fastq file was converted to a fasta file for metagenomics analysis.

**Metagenomic abundance estimation from sequencing data**

Reads mapping to microbial species were identified using HS-BLASTN [22] (hs-blastn-1.0.0), and microbial abundances were estimated using GRAMMy [23](version 1). Specific to SIFT-seq, read-level filtering of contaminants is performed by removing sequenced reads that contain 4 or more cytosines or one methylated CpG dinucleotide (the latter represents unmapped, human-derived molecules). Species-level filtering based on the distribution of mapped reads is carried out by first aligning filtered and unfiltered data sets independently. Cytosine densities of mapping coordinates in both datasets are measured using custom scripts, and their distributions are compared using a Kolmogorov–Smirnov test. Significantly different filtered-unfiltered distributions are further processed (D-statistic > 0.1 and p-value < 0.01). Briefly, filtered datasets whose distribution of cytosines at mapped locations is significantly lower than unfiltered datasets have one read removed, and are re-tested for differences in their distribution. If the distributions are more similar (as measured through the same criteria), it is filtered out. This process is repeated until distributions are no longer significantly different, or if all reads are removed. Read and species-level filtering were performed using custom scripts written in Python.

**Cell-type and Tissue of Origin deconvolution**

cfDNA tissue-of-origin analysis was performed as described by Cheng et al [24] and references from Loyfer et al[25].

**References**

1. Moreno I, Garcia-Grau I, Bau D *et al.* The first glimpse of the endometrial microbiota in early pregnancy. *Am J Obstet Gynecol* 2020;**222**:296–305.

2. Panzer JJ, Romero R, Greenberg JM *et al.* Is there a placental microbiota? A critical review and re-analysis of published placental microbiota datasets. *BMC Microbiol* 2023;**23**:76.

3. Païssé S, Valle C, Servant F *et al.* Comprehensive description of blood microbiome from healthy donors assessed by 16 S targeted metagenomic sequencing. *Transfusion (Paris)* 2016;**56**:1138–47.

4. Castillo DJ, Rifkin RF, Cowan DA *et al.* The Healthy Human Blood Microbiome: Fact or Fiction? *Front Cell Infect Microbiol* 2019;**9**:148.

5. Sterpu I, Fransson E, Hugerth LW *et al.* No evidence for a placental microbiome in human pregnancies at term. *Am J Obstet Gynecol* 2021;**224**:296.e1-296.e23.

6. Kuperman A, Zimmerman A, Hamadia S *et al.* Deep microbial analysis of multiple placentas shows no evidence for a placental microbiome. *BJOG Int J Obstet Gynaecol* 2020;**127**:159–69.

7. Eisenhofer R, Minich JJ, Marotz C *et al.* Contamination in Low Microbial Biomass Microbiome Studies: Issues and Recommendations. *Trends Microbiol* 2019;**27**:105–17.

8. Weyrich LS, Farrer AG, Eisenhofer R *et al.* Laboratory contamination over time during low-biomass sample analysis. *Mol Ecol Resour* 2019;**19**:982–96.

9. Glassing A, Dowd SE, Galandiuk S *et al.* Inherent bacterial DNA contamination of extraction and sequencing reagents may affect interpretation of microbiota in low bacterial biomass samples. *Gut Pathog* 2016;**8**:24.

10. Salter SJ, Cox MJ, Turek EM *et al.* Reagent and laboratory contamination can critically impact sequence-based microbiome analyses. *BMC Biol* 2014;**12**:87.

11. Gaskell M, Guiness J, Sullivan K. Understanding and mitigating the risks that environmental DNA contamination poses to the recovery of forensic evidence from victims and suspects of rape and sexual assault. *J Forensic Leg Med* 2025;**114**:102911.

12. Mzava O, Cheng AP, Chang A *et al.* A metagenomic DNA sequencing assay that is robust against environmental DNA contamination. *Nat Commun* 2022;**13**:4197.

13. Shapiro R, DeFate V, Welcher M. Deamination cytosine derivatives by bisulfite. Mechanism of the reaction. *J Am Chem Soc* 1974;**96**:906–12.

14. Shapiro R, Klein RS. The Deamination of Cytidine and Cytosine by Acidic Buffer Solutions. Mutagenic Implications^*^. *Biochemistry* 1966;**5**:2358–62.

15. Lindahl T, Nyberg B. Heat-induced deamination of cytosine residues in deoxyribonucleic acid. *Biochemistry* 1974;**13**:3405–10.

16. Brian Bushnell. BBMap short read aligner, and other bioinformatic tools.

17. Krueger F, Andrews SR. Bismark: a flexible aligner and methylation caller for Bisulfite-Seq applications. *Bioinformatics* 2011;**27**:1571–2.

18. Song Q, Decato B, Hong EE *et al.* A Reference Methylome Database and Analysis Pipeline to Facilitate Integrative and Comparative Epigenomics. *PLoS ONE* 2013;**8**:e81148.

19. Langmead B, Salzberg SL. Fast gapped-read alignment with Bowtie 2. *Nat Methods* 2012;**9**:357–9.

20. Li H, Handsaker B, Wysoker A *et al.* The Sequence Alignment/Map format and SAMtools. *Bioinformatics* 2009;**25**:2078–9.

21. Magoč T, Salzberg SL. FLASH: fast length adjustment of short reads to improve genome assemblies. *Bioinforma Oxf Engl* 2011;**27**:2957–63.

22. Chen Y, Ye W, Zhang Y *et al.* High speed BLASTN: an accelerated MegaBLAST search tool. *Nucleic Acids Res* 2015;**43**:7762–8.

23. Xia LC, Cram JA, Chen T *et al.* Accurate Genome Relative Abundance Estimation Based on Shotgun Metagenomic Reads. Dias-Neto E (ed.). *PLoS ONE* 2011;**6**:e27992.

24. Cheng AP, Burnham P, Lee JR *et al.* A cell-free DNA metagenomic sequencing assay that integrates the host injury response to infection. *Proc Natl Acad Sci* 2019;**116**:18738–44.

25. Loyfer N, Magenheim J, Peretz A *et al.* A DNA methylation atlas of normal human cell types. *Nature* 2023;**613**:355–64.
